# TOP 2025: An update to the Transparency and Openness Promotion Guidelines

**DOI:** 10.1186/s41073-026-00223-0

**Published:** 2026-08-05

**Authors:** Sean Grant, Katherine S. Corker, David Mellor, Suzanne L. K. Stewart, Evan Mayo-Wilson, Aidan G. Cashin, Malgorzata Lagisz, David Moher, Daniel Umpierre, Virginia Barbour, Stuart Buck, Gary S. Collins, Haley F. Hazlett, Iain Hrynaszkiewicz, Carole J. Lee, Timothy H. Parker, Melissa L. Rethlefsen, Elaine Toomey, Brian A. Nosek

**Affiliations:** 1 College of Education, University of Oregon, Eugene, OR United States; 2 ASAPbio, San Francisco, CA United States; 3 Center for Open Science, Charlottesville, VA United States; 4 Division of Psychology, University of Chester, Chester, United Kingdom; 5 UNC Gillings School of Global Public Health, Chapel Hill, NC USA; 6 School of Population Health, Faculty of Medicine & Health, University of New South Wales, Sydney, Australia; 7 Faculty of Science - Biological Sciences, University of Alberta, Edmonton, Canada; 8 Centre for Journalology, Methodological and Implementation Research Program, Ottawa Hospital Research Institute, Ottawa, Canada; 9 School of Epidemiology and Public Health, University of Ottawa, Ottawa, Canada; 10 Dalla Lana School of Public Health, Institute of Health Policy, Management & Evaluation, University of Toronto, Toronto, Canada; 11 Hospital de Clinicas de Porto Alegre, Universidade Federal do Rio Grande do Sul, Porto Alegre, Brazil; 12 Faculty of Health, Queensland University of Technology, Brisbane, Australia; 13 Good Science Project, Roswell, GA USA; 14 Department of Applied Health Sciences, School of Health Sciences, College of Medicine and Health, University of Birmingham, Birmingham, United Kingdom; 15 Declaration on Research Assessment, Rockville, MD USA; 16 Public Library of Science (PLOS), Cambridge, United Kingdom; 17 Department of Philosophy and Center for an Informed Public, University of Washington, Seattle, United States; 18 Department of Biology, Whitman College, Walla Walla, WA USA; 19 Health Sciences Library & Informatics Center, University of New Mexico, Albuquerque, Mexico; 20 Centre for Health Research Methodology, School of Nursing and Midwifery, University of Galway, Galway, Ireland; 21 Center for Open Science, Charlottesville, Virginia United States of America; 22 University of Virginia, Charlottesville, Virginia United States of America

**Keywords:** Open science, Replication, Reproducibility, Research transparency, TOP Guidelines, Verification

## Abstract

**Background:**

Transparency and openness are core scientific values that enable independent verification of reported research results. The Transparency and Openness Promotion (TOP) Guidelines, first published in 2015 (TOP 2015), provided a flexible framework for journals to design and implement publication standards promoting these values. Over the past decade, widespread use of TOP 2015 contributed to valuable evidence and feedback for refinement.

**Main Text:**

The TOP Advisory Board undertook a comprehensive update of TOP 2015 that went into effect in 2025 (TOP 2025). The update introduces an explicit conceptual framework clarifying its primary objective: improving the verifiability of empirical research claims. Standards are reorganized into three categories (Research Practices, Verification Practices, and Verification Studies) that resolve conceptual and structural limitations of TOP 2015. Research Practices include seven discrete practices implemented in three ways: Disclosed, Shared and Cited, and Certified. Verification Practices introduce standards for computational reproducibility and results transparency, while Verification Studies enumerate empirical research designs and publication formats that support verification of results. Language now centers on researchers and studies (rather than journals) to facilitate adoption by funders, research organizations, preprint servers, evidence curators, and other interest-holders. These changes promote flexible implementation and contextual tailoring.

**Conclusions:**

TOP 2025 retains the core strengths of TOP 2015 while addressing feedback from a decade of implementation and advances in open science. Rather than a universal mandate, it provides a shared, adaptable framework to coordinate the design, implementation, and communication of open policies across interest-holders.

## Background

Transparency and openness are widely regarded as core scientific values [1, 2], in part because they facilitate independent verification of reported research methods and results [3, 4]. Published in 2015, the Transparency and Openness Promotion Guidelines (TOP 2015) provided a common basis for journals and publishers to design and implement eight publication standards aligned with these values [5]. The development of shared standards and terminology aimed to support understanding and communication across study designs and disciplines. The flexible, modular format allowed journals to adopt TOP 2015 as appropriate for their aims, scope, and context by selecting standards and levels of implementation appropriate for the types of research that they publish. For example, journals could implement different standards at different levels of stringency for different study designs. Since its publication, interest-holders have used TOP 2015 across various disciplines. More than 5,000 journal and organizational signatories expressed their support and committed to using TOP 2015 to review their standards [6]. According to Google Scholar, TOP 2015 has been cited over 3,000 times (as of March 2026). Evidence suggests that editors understand and support the underlying principles [7], and journals implementing policies at higher levels of stringency are associated with more transparent empirical research [8].

Over the last decade, interest-holders have identified several opportunities to refine TOP 2015 and to strengthen its dissemination. Experience applying TOP 2015 revealed that its standards and implementation levels were sometimes interpreted inconsistently [9–11]. Variation in how definitions were understood made it challenging for journals and authors to design and interpret policies aligned with TOP 2015 [12]. Moreover, ordinal levels of implementation inadvertently signaled that greater stringency was always preferable, and dissemination efforts sometimes conveyed that all standards were universally applicable [13, 14]. In 2020, the Center for Open Science (COS) introduced the TOP Factor, a separate initiative that rated journal policies based on TOP 2015. Some audiences conflated TOP Factor with TOP 2015. TOP 2015 [5] specified that appropriate standards depend on study design and other factors, whereas TOP Factor provided a simplistic cumulative score across all standards. Additionally, later evidence indicated limitations in the reliability and validity of TOP Factor scores due to challenges in interpreting policy alignment with TOP 2015 standards [9]. These insights highlight the value of clarifying definitions, improving applicability across research designs, and supporting consistent implementation. In response to these issues, we updated TOP 2015 to enhance clarity, flexibility, and alignment with diverse research workflows.

### Updating the TOP guidelines

TOP 2015 was designed to be revised as evidence and experience with its implementation accumulated [5]. To foster community-centered credibility of the TOP Guidelines, COS convened an independent advisory board in 2022 responsible for recommending revisions to the guidelines based on relevant evidence and interest-holder input. The TOP Advisory Board bylaws state that the board shall meet twice a year and that there shall be 5-to-20 members who differ in expertise, discipline, and location. No more than five COS staff and its designees serve in an ex officio (non-voting) capacity. Board members are expected to participate actively in board activities, review agendas and supporting materials before meetings, contribute to subcommittees or special assignments as needed, suggest nominees who can strengthen the board’s work, and help maintain a diverse and representative membership. Additionally, board members may determine education needs and appropriate strategies to achieve them, conduct or suggest potential outreach activities, work with relevant external groups to implement TOP, assist in designing evaluation criteria, and actively solicit community feedback to identify shortcomings and propose revisions. During the update process, the Advisory Board membership included researchers, methodologists, journal editors, and representatives of scholarly infrastructure organizations. Their disciplinary backgrounds spanned the social, behavioral, and biomedical sciences, as well as information science, ecology, and meta-research.

**Fig. 1.**
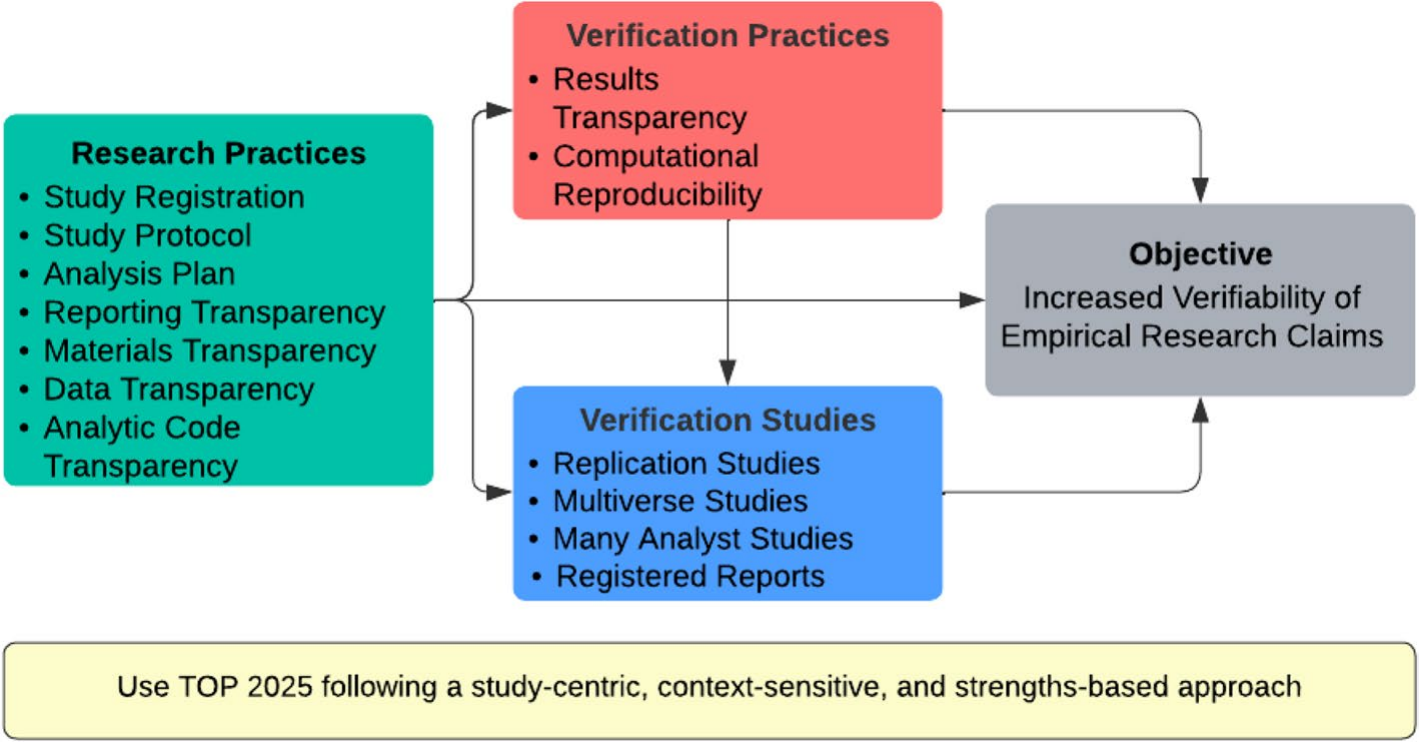
Conceptual Framework for the TOP Guidelines. This figure articulates the primary objective of the TOP Guidelines: improving the verifiability of empirical research claims. Depending on study design, resources, and disciplinary norms, researchers might perform one or more *Research Practices*, each of which results in discrete open research outputs that increase the transparency of empirical research studies. A party independent from the researchers might then perform *Verification Practices*, enabled by one or more Research Practices, to verify that reported results are accurate. *Verification Studies* use specific designs and publication formats to promote verifiability. They are enabled by one or more Research Practices, as well as examining verified results and incorporating Verification Practices. TOP 2025 should be implemented using a study-centric, context-sensitive, and strengths-based approach

The TOP Advisory Board updated TOP 2015 through biannual virtual meetings from 2022 to 2024, seeking interest-holder feedback and relevant meta-scientific literature throughout the update process. After each meeting, a subcommittee drafted and refined proposed changes to TOP 2015 for circulation and discussion with the full board. A preliminary version of the update was posted online for public comment in fall 2024 [15], after which the board incorporated feedback and officially approved proposed changes. COS updated the TOP Guidelines website in 2025 to reflect these changes, replacing TOP 2015 (https://www.cos.io/initiatives/top-guidelines). For the remainder of this manuscript, “guidelines” refers to TOP 2025 as a whole, “framework” refers to the conceptual framework depicted in Figure 1, and “standards” refers to the specific practices and studies described in Table 1..

**Table 1:** Summary of the TOP 2025 Standards. Summary of the recommended standards for Research Practices, Verification Practices, and Verification Studies in TOP 2025. Research Practices can be implemented by researchers, journals, and other organizations, with categories reflecting increasing stringency of implementation requirements. Verification Practices and Verification Studies are activities that institutions can promote. Verification Practices are each enabled by a combination of one or more Research Practices. Verification Studies are empirical research designs and publication formats that can be enabled, incentivized, or required to address verifiability. The current version is maintained on the TOP Guidelines website (https://www.cos.io/initiatives/top-guidelines)

**Research Practices**			
**Practice**	**Disclosed**	**Shared and Cited**	**Certified**
Study Registration	Researchers stated whether a study was registered—and, if so, where and when it was registered.	Researchers registered the study and cited the registration.	A party independent from the researchers certified that the study was registered at an appropriate time and the registration was complete per best practice for the study design.
Study Protocol	Researchers stated whether the study protocol is available—and, if so, where and when it was shared.	Researchers publicly shared the study protocol and cited the protocol.	A party independent from the researchers certified that the study protocol was shared at an appropriate time and the study protocol was complete per best practice for the study design.
Analysis Plan	Researchers stated whether the analysis plan is available—and, if so, where and when it was shared.	Researchers publicly shared the analysis plan and cited the analysis plan.	A party independent from the researchers certified that the analysis plan was shared at an appropriate time and the analysis plan was complete per best practice for the study design.
Reporting Transparency	Researchers stated whether they used a reporting guideline—and, if so, which guideline.	Researchers publicly shared the completed reporting guideline checklist and cited the reporting guideline.	A party independent from the researchers certified that the researchers adhered to the appropriate reporting guideline for the study design.
Materials Transparency	Researchers stated whether materials are available—and, if so, where.	Researchers cited materials deposited in a trusted repository by themselves or others.	A party independent from the researchers certified that materials were deposited and documented per best practice for the type of materials.
Data Transparency	Researchers stated whether data are available—and, if so, where.	Researchers cited data deposited in a trusted repository by themselves or others.	A party independent from the researchers certified that data were deposited with metadata per best practice for the type of data.
Analytic Code Transparency	Researchers stated whether analytic code is available—and, if so, where.	Researchers cited analytic code deposited in a trusted repository by themselves or others.	A party independent from the researchers certified that analytic code was deposited and documented per relevant best practice.
**Verification Practices**			
**Practice**	**Definition**		
Results Transparency	A party independent from the researchers verified that results have not been reported selectively based on the nature of the findings. To verify, the independent party can check that the study registration, protocol, and analysis plan match the final report–and the final report acknowledges any deviations.		
Computational Reproducibility	A party independent from the researchers verified that reported results reproduce using the same data and following the same computational procedures. To verify, the independent party can check that they obtain the same results using data and code deposited in a trusted repository.		
**Verification Studies**			
**Study Type**	**Definition**		
Replication	A study that aims to provide diagnostic evidence about claims from a prior study by repeating the original study procedures in a new sample.		
Registered Report	A registered study in which a study protocol and analysis plan are peer reviewed—and the study is accepted in-principle by a publication outlet—before the research is undertaken (i.e., two-stage peer review with in-principle acceptance).		
Multiverse	A study in which a single research team examines the research question of interest across different, reasonable choices for processing and analyzing the same data.		
Many Analysts	A study in which independent analysis teams conduct plausible alternative analyses of a research question on the same dataset.		

### A conceptual framework for the TOP guidelines

To address questions about its scope, TOP 2025 includes a conceptual framework (Fig. 1) that clarifies its primary objective—improving the verifiability of empirical research claims—and guides its organization and recommended applications. Although TOP 2015 shared these goals implicitly, lack of an explicit conceptual framework contributed to different interpretations of its purpose and scope. For example, the TOP Advisory Board received interest-holder requests that were out of scope for TOP 2025, such as standards for open publishing [16] and inclusive science [17]. Although members of the TOP Advisory Board also promote these aspects of open science [18], they fall outside the primary objective of TOP.

### Reorganized standards

TOP 2025 is organized around *Research Practices, Verification Practices,* and *Verification Studies* (Table 1.). The reorganization addresses several limitations of TOP 2015. Standards in TOP 2015 included a mix of discrete research practices (e.g., data sharing), combinations of research practices (e.g., computational reproducibility), and specific types of studies and publications (e.g., replication). Terms used to describe levels of implementation in TOP 2015—disclose, require, verify—did not apply consistently across the standards. Moreover, some standards were intertwined; for instance, the most stringent implementations of the Data Transparency standard and the Code Transparency standard each required computational reproducibility; they were not independent standards because code is not sufficient to reproduce findings without data. These conceptual and organizational problems contributed to difficulties interpreting and applying the standards, particularly at higher levels of implementation [9].

*Research Practices*. TOP 2025 includes standards for seven discrete practices that are related closely to the TOP 2015 standards. Each of the included Research Practices results in discrete open research *outputs* that facilitate verification of reported research results [19, 20]. Some of these outputs can function as both shareable products of research practices as well as inputs to the research process. In TOP 2025, “output” refers to a discrete, shareable product that results from performing a Research Practice, regardless of when in the research workflow it is created. These are (1) study registration, (2) study protocols, (3) analysis plans, (4) reporting transparency, (5) materials transparency, (6) data transparency, and (7) code transparency. TOP 2025 uses the term “study” broadly to refer to a discrete empirical research endeavor that aims to answer one or more research questions. While not the primary reasons for including them in TOP 2025, these Research Practices also facilitate other benefits such as reuse and extension of previous research.

TOP 2025 retains three categories of implementation for Research Practices: Disclosed, Shared and Cited, and Certified. *Disclosure* requires that researchers state whether they performed a practice—and, if so, state the location of the resultant research output [21]. Disclosure requirements minimize implementation barriers while signaling that open research outputs are valued [5]. *Sharing and Citation* requires that researchers perform a practice, make the resultant open research output available publicly, and cite the output in the references section with a permanent unique identifier. These requirements apply to both primary studies (e.g., clinical trials) and secondary research outputs (e.g., studies using a publicly available dataset). For Data Transparency, researchers must provide a citation to the location of data in a repository, which might be data they collected or a dataset deposited by someone else. *Certification* further requires that a party independent from the researchers confirm that sharing was done according to best practices for a given study design. For example, certification of study registration for a randomized trial might involve a third party confirming that the trial was registered before enrollment of the first participant [22] and includes information consistent with established standards such as the WHO Trial Registration Data Set [23]. Certification involves the most resources, which can present barriers to implementation. TOP 2025 calls for users to make appropriate exceptions to policy requirements, which might relate to data sovereignty [24], ethical and human rights issues, legal and proprietary concerns, and limited availability of necessary resources.

*Verification Practices*. In addition to standards focused on research practices, TOP 2025 introduces two standards for verifying that reported results accurately describe what was found. *Computational Reproducibility* corresponds with Level 3 for the Materials, Data, and Code Transparency standards in TOP 2015. It requires that a party independent from the researchers confirm that reported results can be obtained using the data and following the same computational procedures described in the report. *Results Transparency* requires that a party independent from the researchers confirm that results have not been reported selectively based on the nature of the findings (e.g., the statistical significance, direction, or magnitude of estimates). An independent party might compare findings in a publication (that follows a relevant reporting guideline) with research outputs created before the study was conducted (e.g., study registration, study protocol, analysis plan).

*Verification Studies*. TOP 2025 also includes four types of empirical research designs and publication formats that support verification of results. In TOP 2015, *Replication* was the only empirical research design and *Registered Reports* [25] was the only publication format (i.e., Replication standard Level 3). In TOP 2025, *Replication* has been retained and *Registered Reports* is no longer subsumed under it. Two additional Verification Studies have gained prominence since TOP 2015: *Multiverse* [26] and *Many Analysts* [27] studies. As examples, the first demonstration of a multiverse study examined the effect of fertility on religiosity and political attitudes across combinations of reasonable data processing decisions (e.g., how to determine fertility status, which participants to exclude, and how to operationalize relationship status), showing that conclusions varied substantially depending on these choices [26]. The first demonstration of a many analyst study involved 61 analysts using the same dataset to examine whether soccer referees are more likely to give red cards to dark-skin-toned players than to light-skin-toned players [28]. Listing these designs and formats in TOP 2025 is not meant to suggest they are exhaustive, nor preclude the addition of other designs and formats in future updates.

### Revised standards

TOP 2025 revises guidance about several Research Practices based on feedback from cross-disciplinary experiences using TOP 2015. First, the previous Citation standard—which extended article citation norms to other research outputs—has been integrated into the Shared and Cited implementation category for *all* Research Practices in TOP 2025. Second, the Design and Analysis Transparency standard in TOP 2015 has been relabeled “Reporting Transparency” to clarify the purpose of this standard and its implementation through the use of reporting guidelines [29]. Lastly, TOP 2025 added Study Protocols as a standard. TOP 2015 used the term “preregistration” to describe a combination of discrete practices (i.e., Study Registration, sharing Study Protocols, and sharing Analysis Plans) and their timing (i.e., “pre-” implying that practices must be performed before some unspecified event). To reduce ambiguity and to improve implementation, TOP 2025 disaggregates “preregistration” by requiring descriptions of which discrete outputs were created and shared in relation to specific study activities [30]. Study Registration involves entering a time-stamped, publicly available record about a study in a register that assigns a unique and permanent study identifier [31]. Certifying this practice includes assessing the information contained in a registration and comparing registration timing with the timings of key study events—such as data access in secondary research [32] or enrollment of the first participant in a clinical trial [22]. Study Protocols and Analysis Plans provide more comprehensive information about planned methods and analyses [30]; they may be in the same document or different documents, shared as preprints or journal articles, included with study registrations, and/or made publicly available prospective to different study events.

### Potential applications

TOP 2025 remains applicable to journal policies and instructions to authors. Journals can use TOP 2025 to design policies on Research and Verification Practices in their instructions to authors, editors, and reviewers. TOP 2025 might also help journals develop procedures to support transparency and openness. With the support of publishers, journals might incorporate disclosures about Research and Verification Practices into their manuscript submission systems and article templates [12]. For journals that implement standards with greater stringency, Research and Verification Practices might be integrated into editorial and peer review procedures. To recognize the value of Verification Studies, journals could list them in their instructions to authors and specify the conditions under which these studies would be considered and published [25].

That said, language for standards and implementation in TOP 2025 focuses on researchers and studies (rather than journals). Research-centric language allows policies to be tailored by study design, context, and use by diverse interest-holders with interests in the verifiability of empirical research claims [33, 34]. For example, preprint servers could implement policies and procedures on Research and Verification Practices. Funders could use TOP 2025 to include Research and Verification Practices in grant requirements as appropriate for targeted disciplines and study designs, and they could provide dedicated funding for Verification Studies of existing empirical research [35]. Organizations that perform research could use TOP 2025 to inform tailored approaches for rewarding the use of Research Practices, Verification Practices, and Verification Studies as part of hiring, promotion, and tenure [36]. Universities, libraries, and other institutions that educate research students and offer research training could use TOP 2025 to design curricula, training, guidelines, and infrastructure [37]. Research societies could use TOP 2025 to inform awards and other recognitions [38]. Organizations that curate research evidence could use TOP 2025 to design assessment criteria [39]. This shared framework could facilitate coordinated implementation across interest-holders [2, 40]. COS has provided examples of how TOP 2025 can be used by organizations with their various roles in the scholarly community [41].

### Critiques and challenges

Several critiques of TOP have offered valuable insights that shaped this update. First, some have perceived TOP as narrowly defining research quality, or conflating transparency and openness with other requirements for rigorous and trustworthy research. TOP 2025 continues to acknowledge that computational irreproducibility and selective reporting facilitated by closed workflows remain important challenges [42], while also recognizing that the relevance of these issues varies across disciplines [43]. Accordingly, the primary application of TOP 2025 is supporting the design of policies that promote practices, study designs, and publication formats addressing these concerns. We also note that the ability to verify reported results is only one indicator of credibility [44, 45]. TOP 2025 is not intended to suggest that verifiability is the most important determinant of research quality [46]. Notably, both strong and weak research can be conducted transparently, as transparency is not synonymous with nor a substitute for methodological rigor [47–49].

Second, TOP has been critiqued for privileging certain disciplines and methodologies. TOP 2015 was developed primarily (though not exclusively) by social and behavioral scientists concerned about misuse of null-hypothesis significance testing [13, 50–52], selective reporting, and computational irreproducibility in published experimental research [5, 53]. As a result, the full set of TOP standards is most directly applicable to quantitative studies and other designs that test questions or hypotheses about potentially generalizable knowledge. Some standards may be less applicable or less important for other epistemologies, study designs, or phenomena of inquiry. Decisions about applicability should therefore rest with disciplinary experts, and experts in different study designs, who can use the modular structure of TOP 2025 to tailor its implementation. TOP 2025 is intended to support contextual decision-making rather than mandate uniformity. Users should design and implement policies that are appropriate for their contexts, practical given available resources, and aligned with community readiness and norms. For example, a journal might require (i) Disclosure for Data Transparency, (ii) Share and Cite for Code Transparency, and (iii) Certification for Study Registration only for randomized trials. Another journal might also decide that the framework is not applicable to the research it publishes. Organizational policies may evolve over time (including possibly through de-implementation) as experience, evidence, and best practice guidance advance.

In addition to these critiques, several broader challenges affect TOP 2025 dissemination and implementation. First, standards can create incentives for performative rather than substantive compliance [54, 55]. To reduce the likelihood of quantitative scoring based on TOP 2025, ordinal implementation levels for Research Practices were replaced with categorical classifiers, and COS has discontinued the TOP Factor. Second, Artificial Intelligence (AI) presents both opportunities and challenges for the implementation of open science standards. AI has the potential to support transparency and openness by improving the discoverability of research outputs [56], automating verification of computational reproducibility [57], assisting with compliance checks for study registrations [58], and improving adherence to reporting guidelines (74). At the same time, emerging evidence suggests that AI might be used to generate large numbers of papers using publicly available data [59–62], raising questions about whether open science policies could inadvertently facilitate proliferation of low-quality publications. As consensus develops on how to address AI-related issues, future updates to TOP may need to refine standards and implementation guidance accordingly [63]. Lastly, dissemination of TOP 2025 is occurring amidst concerns about the misuse of open science rhetoric as a pretext to attack science [64–67]. We reaffirm that open science should not be co-opted in bad faith, and that transparency and openness should strengthen science and support evidence-based policy [68, 69]. Taken together, these challenges underscore the importance of purposefully disseminating TOP 2025 through context-sensitive, study-centric, and strengths-based approaches to support implementation aligned with its intent [70].

### Continued evaluation and revision

The authors and current TOP Advisory Board will revise these guidelines as implementation experience and evidence accumulates. They will also support implementation by providing training and working with journals and professional societies. Additionally, to assist implementers in using TOP 2025 to design organizational policies and meta-research, COS is maintaining a support team, updating its detailed implementation guidance, and providing worked examples of TOP 2025 in action for specific organizational contexts on its blog. The authors, current Advisory Board, and COS welcome feedback based on first-hand implementation experiences and meta-research, including constructive criticisms from diverse meta-scientific perspectives [71].

## Conclusions

TOP 2025 updates and replaces the original TOP Guidelines published in 2015. It retains core strengths while addressing feedback, introduces an explicit conceptual framework, and improves organization. Revisions clarify and streamline guidance, providing a shared framework that interest-holders can use to communicate their policies and coordinate efforts to improve the verifiability of empirical research claims.

## Data Availability

Summaries of advisory board meeting minutes, interest-holder feedback, and previous drafts of TOP 2025 can be found on the Open Science Framework: (https://osf.io/ews92).

## References

[1] UNESCO. “UNESCO recommendation on open science.” UNESCO; 2021. 10.54677/MNMH8546.

[2] Open Science by Design: Realizing a Vision for 21st Century Research (National Academies Press, Washington, D.C., 2018; https://www.nap.edu/catalog/25116). 30212065

[3] Arrison T, Saunders J, Kameyama E, editors. Developing a toolkit for fostering open science practices: proceedings of a workshop. Washington, D.C.: National Academies Press; 2021. 34784154

[4] Reproducibility and Replicability in Science (National Academies Press, Washington, D.C., 2019; https://www.nap.edu/catalog/25303). 31596559

[5] Nosek BA, Alter G, Banks GC, Borsboom D, Bowman SD, Breckler SJ, et al. Promoting an open research culture. Science. 2015;348:1422–5. 26113702 10.1126/science.aab2374 PMC4550299

[6] Center for Science, TOP Guidelines. https://www.cos.io/initiatives/top-guidelines.

[7] Naaman K, Grant S, Kianersi S, Supplee L, Henschel B, Mayo-Wilson E. Exploring enablers and barriers to implementing the Transparency and Openness Promotion Guidelines: a theory-based survey of journal editors. R Soc Open Sci. 2023;10:221093. 36756061 10.1098/rsos.221093 PMC9890101

[8] L. Dudda, E. Kormann, M. Kozula, N. J. DeVito, T. Klebel, A. P. M. Dewi, R. Spijker, I. Stegeman, V. V. den Eynden, T. Ross-Hellauer, M. Leeflang, Open Science interventions to improve reproducibility and replicability of research: a scoping review preprint. OSF [Preprint] (2024). 10.31222/osf.io/a8rmu. 10.1098/rsos.242057 PMC11979971 40206851

[9] Kianersi S, Grant S, Naaman K, Henschel B, Mellor DT, Apte S, et al. Evaluating implementation of the Transparency and Openness Promotion Guidelines: reliability of instruments to assess journal policies, procedures, and practices. Adv Methods Pract Psychol Sci. 2023;6:1–21.

[10] Toomey E, Coyne R, Derksen C, Grant SP, Jones CM, Kijowska M, et al. Adoption of the Transparency and Openness Promotion (TOP) guidelines within health psychology and behavioural medicine journal policies: a cross-sectional study. Health Psychol Rev. 2025;19:763–80. 40513012 10.1080/17437199.2025.2516010

[11] Hansford HJ, Cashin AG, Bagg MK, Wewege MA, Ferraro MC, Kianersi S, et al. Feasibility of an audit and feedback intervention to facilitate journal policy change towards greater promotion of transparency and openness in sports science research. Sports Med. 2022;8:101. 10.1186/s40798-022-00496-x PMC9357245 35932429

[12] Mayo-Wilson E, Grant S, Supplee L, Kianersi S, Amin A, DeHaven A, et al. Evaluating implementation of the Transparency and Openness Promotion (TOP) guidelines: the TRUST process for rating journal policies, procedures, and practices. Res Integr Peer Rev. 2021;6:9. 34078479 10.1186/s41073-021-00112-8 PMC8173977

[13] Lash TL. Getting over TOP. Epidemiology. 2022;33:1. 34847082 10.1097/EDE.0000000000001424

[14] C. Vinatier, A. P. M. Dewi, G. Freyermuth, M. Millour, M. Scheidecker, F.-J. Arnault, M. Acher, V. Nachev, T. L. Weissgerber, N. J. DeVito, G. Dumont, G. Gopalakrishna, G. L. B. Lyan, I. Stegeman, M. M. G. Leeflang, F. Naudet, Monitoring of Open Science practices: a survey of 10 major medical journals. medRxiv [Preprint] (2025). 10.1101/2025.11.26.25341061.

[15] S. Grant, K. S. Corker, D. T. Mellor, S. L. K. Stewart, A. G. Cashin, M. Lagisz, E. Mayo-Wilson, D. Moher, D. Umpierre, V. Barbour, S. Buck, G. Collins, H. Hazlett, I. Hrynaszkiewicz, C. J. Lee, T. H. Parker, M. L. Rethlefsen, E. Toomey, B. A. Nosek, TOP 2025: An Update to the Transparency and Openness Promotion Guidelines. MetaArXiv nmfs6_v1 [Preprint] (2024). 10.31222/osf.io/nmfs6.

[16] Vazire S. The next chapter for Psychological Science. Psychol Sci. 2023. 10.1177/09567976231221558. 10.1177/09567976231221558 38150595

[17] Ledgerwood A, Hudson STJ, Lewis NA, Maddox KB, Pickett CL, Remedios JD, et al. The pandemic as a portal: reimagining psychological science as truly open and inclusive. Perspect Psychol Sci. 2022;17:937–59. 35235485 10.1177/17456916211036654

[18] Silverstein P, Elman C, Montoya A, McGillivray B, Pennington CR, Harrison CH, et al. A guide for social science journal editors on easing into open science. Res Integr Peer Rev. 2024;9:2. 38360805 10.1186/s41073-023-00141-5 PMC10870631

[19] Stodden V, Seiler J, Ma Z. An empirical analysis of journal policy effectiveness for computational reproducibility. Proc Natl Acad Sci U S A. 2018;115:2584–9. 29531050 10.1073/pnas.1708290115 PMC5856507

[20] TARG Meta-Research Group and Collaborators. Discrepancy review: a feasibility study of a novel peer review intervention to reduce undisclosed discrepancies between registrations and publications. R Soc Open Sci. 2022;9:220142. 35911195 10.1098/rsos.220142 PMC9326291

[21] The TOP Statement Working Group, Making Science Transparent By Default; Introducing the TOP Statement. OSF Preprints [Preprint] (2018). 10.31219/osf.io/sm78t.

[22] De Angelis C, Drazen JM, Frizelle FA, Haug C, Hoey J, Horton R, et al. Clinical trial registration: a statement from the International Committee of Medical Journal Editors. N Engl J Med. 2004;351:1250–1. 15356289 10.1056/NEJMe048225

[23] Sim I, Chan A-W, Gülmezoglu AM, Evans T, Pang T. Clinical trial registration: transparency is the watchword. Lancet. 2006;367:1631–3. 16714166 10.1016/S0140-6736(06)68708-4

[24] Carroll SR, Garba I, Figueroa-Rodríguez OL, Holbrook J, Lovett R, Materechera S, et al. The CARE principles for Indigenous data governance. Data Sci J. 2020. 10.5334/dsj-2020-043.

[25] Chambers CD, Tzavella L. The past, present and future of registered reports. Nat Hum Behav. 2022;6:29–42. 34782730 10.1038/s41562-021-01193-7

[26] Steegen S, Tuerlinckx F, Gelman A, Vanpaemel W. Increasing transparency through a multiverse analysis. Perspect Psychol Sci. 2016;11:702–12. 27694465 10.1177/1745691616658637

[27] Aczel B, Szaszi B, Nilsonne G, van den Akker OR, Albers CJ, van Assen MA, et al. Consensus-based guidance for conducting and reporting multi-analyst studies. eLife. 2021;10:e72185. 34751133 10.7554/eLife.72185 PMC8626083

[28] R. Silberzahn, E. L. Uhlmann, D. P. Martin, P. Anselmi, F. Aust, E. Awtrey, Š. Bahník, F. Bai, C. Bannard, E. Bonnier, R. Carlsson, F. Cheung, G. Christensen, R. Clay, M. A. Craig, A. Dalla Rosa, L. Dam, M. H. Evans, I. Flores Cervantes, N. Fong, M. Gamez-Djokic, A. Glenz, S. Gordon-McKeon, T. J. Heaton, K. Hederos, M. Heene, A. J. Hofelich Mohr, F. Högden, K. Hui, M. Johannesson, J. Kalodimos, E. Kaszubowski, D. M. Kennedy, R. Lei, T. A. Lindsay, S. Liverani, C. R. Madan, D. Molden, E. Molleman, R. D. Morey, L. B. Mulder, B. R. Nijstad, N. G. Pope, B. Pope, J. M. Prenoveau, F. Rink, E. Robusto, H. Roderique, A. Sandberg, E. Schlüter, F. D. Schönbrodt, M. F. Sherman, S. A. Sommer, K. Sotak, S. Spain, C. Spörlein, T. Stafford, L. Stefanutti, S. Tauber, J. Ullrich, M. Vianello, E.-J. Wagenmakers, M. Witkowiak, S. Yoon, B. A. Nosek, Many Analysts, One Data Set: Making Transparent How Variations in Analytic Choices Affect Results. Advances in Methods and Practices in Psychological Science. 2018;1:337–356.

[29] Simera I, Moher D, Hirst A, Hoey J, Schulz KF, Altman DG. Transparent and accurate reporting increases reliability, utility, and impact of your research: reporting guidelines and the EQUATOR Network. BMC Med. 2010;8:24. 20420659 10.1186/1741-7015-8-24 PMC2874506

[30] Mayo-Wilson E, Grant S, Corker KS, Moher D. Consistent and precise description of research outputs could improve implementation of open science. Adv Methods Pract Psychol Sci. 2025;8:25152459251375445.

[31] Rice DB, Moher D. Curtailing the use of preregistration: a misused term. Perspect Psychol Sci. 2019;14:1105–8. 31449761 10.1177/1745691619858427

[32] Van den Akker O, Weston S, Campbell L, Chopik B, Damian R, Davis-Kean P, Bakker M. Preregistration of secondary data analysis: A template and tutorial. Meta-psychology. 2021;5:2625. 10.15626/MP.2020.2625.

[33] B. A. Nosek, L. C. Shaw, T. M. Errington, N. Pfeiffer, D. T. Mellor, R. E. B. Iii, A. Rice, D. M. Litherland, Center for Open Science: Strategic Plan. OSF [Preprint] (2017). 10.31219/osf.io/x2w9h.

[34] Reproducibility and transparency. what’s going on and how can we help. Nat Commun. 2025;16:1082. 39870612 10.1038/s41467-024-54614-2 PMC11772749

[35] Mellor D. Improving norms in research culture to incentivize transparency and rigor. Educ Psychol. 2021;56:122–31.

[36] Moher D, Naudet F, Cristea IA, Miedema F, Ioannidis JPA, Goodman SN. Assessing scientists for hiring, promotion, and tenure. PLoS Biol. 2018;16:e2004089. 29596415 10.1371/journal.pbio.2004089 PMC5892914

[37] Tackett JL, Brandes CM, Dworak EM, Shields AN. Bringing the (pre)registration revolution to graduate training. Can Psychol. 2020;61:299–309.

[38] Lagisz M, Rutkowska J, Aich U, Ross RM, Santana MS, Wang J, et al. “Best paper” awards lack transparency, inclusivity, and support for Open Science. PLoS Biol. 2024;22:e3002715. 39042591 10.1371/journal.pbio.3002715 PMC11265724

[39] Mayo-Wilson E, Grant S, Supplee LH. Clearinghouse standards of evidence on the transparency, openness, and reproducibility of intervention evaluations. Prev Sci. 2022;23:774–86. 34357509 10.1007/s11121-021-01284-x PMC9283145

[40] Rasti S, Vaesen K, Lakens D. A systemic approach to better coordination in science. Nat Hum Behav. 2025;9:1762–4. 40595430 10.1038/s41562-025-02260-z

[41] D. Mellor, M. Zaringhalam, From Policy to Practice: COS’s Commitment to Applying the Transparency and Openness Promotion (TOP) Guidelines (2025). https://www.cos.io/blog/from-policy-to-practice-coss-commitment-to-applying-the-transparency-and-openness-promotion-top-guidelines.

[42] Curry S, Mercado-Lara E, Arechavala-Gomeza V, Begley CG, Bernard C, Bernard R, et al. Ending publication bias: A values-based approach to surface null and negative results. PLOS Biology. 2025;23:e3003368. 40991668 10.1371/journal.pbio.3003368 PMC12459790

[43] Bartoš F, Maier M, Wagenmakers E-J, Nippold F, Doucouliagos H, Ioannidis JPA, et al. Footprint of publication selection bias on meta-analyses in medicine, environmental sciences, psychology, and economics. Res Synth Methods. 2024;15:500–11. 38327122 10.1002/jrsm.1703

[44] Farrington DP. Methodological quality standards for evaluation research. Ann Am Acad Polit Soc Sci. 2003;587:49–68.

[45] B. A. Nosek, D. Allison, K. H. Jamieson, M. McNutt, A. B. Nielsen, S. M. Wolf, A Framework for Assessing the Trustworthiness of Research Findings. MetaArXiv jw6fz_v1 [Preprint] (2024). 10.31222/osf.io/jw6fz. 10.1073/pnas.2536736123 PMC12890942 41632849

[46] Guyatt GH, Oxman AD, Vist GE, Kunz R, Falck-Ytter Y, Alonso-Coello P, et al. GRADE: an emerging consensus on rating quality of evidence and strength of recommendations. BMJ. 2008;336:924–6. 18436948 10.1136/bmj.39489.470347.AD PMC2335261

[47] Vazire S. Why should you trust research published in Psychological Science? Psychol Sci. 2025;36:311–5. 40334644 10.1177/09567976251336246

[48] Auspurg K. Robustness is better assessed with a few thoughtful models than with billions of regressions. Proceedings of the National Academy of Sciences. 2025;122:e2521917122. 10.1073/pnas.2521917122 PMC12582322 41115196

[49] Auspurg K, Brüderl J. Toward a more credible assessment of the credibility of science by many-analyst studies. Proc Natl Acad Sci U S A. 2024;121:e2404035121. 39236231 10.1073/pnas.2404035121 PMC11420151

[50] Lash TL. Declining the Transparency and Openness Promotion Guidelines. Epidemiology. 2015;26:779. 26348161 10.1097/EDE.0000000000000382

[51] Lash TL. The Harm Done to Reproducibility by the Culture of Null Hypothesis Significance Testing. Am J Epidemiol. 2017;186:627–35. 28938715 10.1093/aje/kwx261

[52] Lash TL, Collin LJ, Van Dyke ME. The replication crisis in epidemiology: Snowball, Snow Job, or Winter Solstice? Curr Epidemiol Rep. 2018;5:175–83. 33907664 10.1007/s40471-018-0148-x PMC8075285

[53] Simmons JP, Nelson LD, Simonsohn U. False-positive psychology: undisclosed flexibility in data collection and analysis allows presenting anything as significant. Psychol Sci. 2011;22:1359–66. 22006061 10.1177/0956797611417632

[54] Fire M, Guestrin C. Over-optimization of academic publishing metrics: observing Goodhart’s Law in action. Gigascience. 2019;8:giz053. 31144712 10.1093/gigascience/giz053 PMC6541803

[55] Jüni P, Witschi A, Bloch R, Egger M. The Hazards of Scoring the Quality of Clinical Trials for Meta-analysis. JAMA. 1999;282:1054–60. 10493204 10.1001/jama.282.11.1054

[56] L. Cadwallader, I. Hrynaszkiewicz, P. Sarin, T. Vines, Measuring research data reuse in scholarly publications using generative artificial intelligence: Open Science Indicator development and preliminary results, arXiv.org. 2026. https://arxiv.org/abs/2604.28061v1.

[57] D. Nüst, S. J. Eglen, CODECHECK: an Open Science initiative for the independent execution of computations underlying research articles during peer review to improve reproducibility. F1000Research [Preprint] (2021). 10.12688/f1000research.51738.2. 10.12688/f1000research.51738.1 PMC8311796 34367614

[58] M. Zeng, S. Liu, D. P. Clark, S. McDonald, E. Mayo-Wilson, X. Ying, J. Menke, M. Lan, L. Jiang, K. Ninan, J.-P. Oberste, J. E. McKenzie, H. Kilicoglu, M. J. Page, Artificial intelligence tools for automating assessments of reporting guideline adherence: a protocol for a systematic review. F1000Research [Preprint] (2026). 10.12688/f1000research.179775.1.

[59] Suchak T, Aliu AE, Harrison C, Zwiggelaar R, Geifman N, Spick M. Explosion of formulaic research articles, including inappropriate study designs and false discoveries, based on the NHANES US national health database. PLoS Biol. 2025;23:e3003152. 40338847 10.1371/journal.pbio.3003152 PMC12061153

[60] M. Spick, A. Onoja, C. Harrison, S. Stender, J. Byrne, N. Geifman, Quantifying new threats to health and biomedical literature integrity from rapidly scaled publications and problematic research. medRxiv [Preprint] (2025). 10.1101/2025.07.07.25331008. 10.1016/j.jclinepi.2026.112203 PMC13178217 41740900

[61] Stender S, Gellert-Kristensen H, Smith GD. Reclaiming Mendelian randomization from the deluge of papers and misleading findings. Lipids Health Dis. 2024;23:286. 39244551 10.1186/s12944-024-02284-w PMC11380209

[62] B. Scancar, J. A. Byrne, D. Causeur, A. G. Barnett, Revealing the Paper Mill Iceberg: AI-Based Screening of Cancer Research Publications. bioRxiv [Preprint] (2025). 10.1101/2025.08.29.673016.

[63] Sabel B, Larhammar D. Reformation of science publishing: the Stockholm Declaration. R Soc Open Sci. 2025;12:251805. 41200241 10.1098/rsos.251805 PMC12586959

[64] Garisto D. Trump order gives political appointees vast powers over research grants. Nature. 2025;644:585–6. 40781565 10.1038/d41586-025-02557-z

[65] Lewandowsky S. Trojan gold: new US “standard” is another veiled attack on science. Science. 2025;390:eaeb9857. 41100613 10.1126/science.aeb9857

[66] Fernandez F, Hutchens N. Restrictions on US academic freedom affect science everywhere. Nat Hum Behav. 2025;9:1303–4. 40490509 10.1038/s41562-025-02248-9

[67] Goldman G, Chenoweth E. Scientists’ role in defending democracy. Science. 2025;389:667–667. 40811528 10.1126/science.aea9328

[68] Gleick PH. More US scientists must speak out. Nat Hum Behav. 2025;9:1305–6. 40456939 10.1038/s41562-025-02240-3

[69] Center for Open Science, COS Statement on “Restoring Gold Standard Science” Executive Order (2025). https://www.cos.io/about/news/cos-statement-on-restoring-gold-standard-science-executive-order.

[70] Santana C. The value of openness in open science. Can J Philos. 2024;54:251–65.

[71] M. Rubin, What is critical metascience and why is it important? PsyArXiv 4tpk8_v2 [Preprint] (2025). 10.31234/osf.io/4tpk8_v2.

